# S100B Engages RAGE or bFGF/FGFR1 in Myoblasts Depending on Its Own Concentration and Myoblast Density. Implications for Muscle Regeneration

**DOI:** 10.1371/journal.pone.0028700

**Published:** 2012-01-20

**Authors:** Francesca Riuzzi, Guglielmo Sorci, Sara Beccafico, Rosario Donato

**Affiliations:** Department of Experimental Medicine and Biochemical Sciences and Istituto Interuniversitario di Miologia, University of Perugia, Perugia, Italy; Istituto Dermopatico dell'Immacolata, Italy

## Abstract

In high-density myoblast cultures S100B enhances basic fibroblast growth factor (bFGF) receptor 1 (FGFR1) signaling via binding to bFGF and blocks its canonical receptor, receptor for advanced glycation end-products (RAGE), thereby stimulating proliferation and inhibiting differentiation. Here we show that upon skeletal muscle injury S100B is released from myofibers with maximum release at day 1 post-injury in coincidence with satellite cell activation and the beginning of the myoblast proliferation phase, and declining release thereafter in coincidence with reduced myoblast proliferation and enhanced differentiation. By contrast, levels of released bFGF are remarkably low at day 1 post-injury, peak around day 5 and decline thereafter. We also show that in low-density myoblast cultures S100B binds RAGE, but not bFGF/FGFR1 thereby simultaneously stimulating proliferation via ERK1/2 and activating the myogenic program via p38 MAPK. Clearance of S100B after a 24-h treatment of low-density myoblasts results in enhanced myotube formation compared with controls as a result of increased cell numbers and activated myogenic program, whereas chronic treatment with S100B results in stimulation of proliferation and inhibition of differentiation due to a switch of the initial low-density culture to a high-density culture. However, at relatively high doses, S100B stimulates the mitogenic bFGF/FGFR1 signaling in low-density myoblasts, provided bFGF is present. We propose that S100B is a danger signal released from injured muscles that participates in skeletal muscle regeneration by activating the promyogenic RAGE or the mitogenic bFGF/FGFR1 depending on its own concentration, the absence or presence of bFGF, and myoblast density.

## Introduction

The regenerative capacity of skeletal muscle tissue resides in satellite cells, the quiescent adult stem cells located between the sarcolemma of the myofiber and the basal lamina [1], [2]. In case of intense physical exercise, trauma or systemic disorders such as infectious diseases, cancer and chronic inflammatory diseases, satellite cells become activated exiting their quiescence status, emigrating and proliferating. Subsequently, activated satellite cells stop proliferating and differentiate into fusion-competent myocytes that form multinucleated syncytia called myotubes, the immediate precursors of myofibers. Proliferation of activated satellite cells is a fundamental step of the muscle regeneration process serving the function of expanding the population of myoblasts and of reconstituting the pool of reserve satellite cells. Conceivably, interference with any one of the above steps will result in defective muscle regeneration capacity. Thus, stimuli and/or conditions that keep satellite cells proliferating will delay or impede muscle regeneration because proliferation and differentiation are mutually exclusive. On the other hand, inhibition of proliferation reduces myoblast numbers and, hence, the number and size of regenerated myofibers as well as the satellite cell reserve pool, and precocious myoblast differentiation results in the formation of (a reduced number of) thin myofibers.

The regeneration of injured muscle tissue requires an ordered activation/inactivation of transcription factors, of which some are muscle-specific (i.e., Myf5, MyoD, myogenin and MRF4) while others (e.g., Pax7, NF-κB, β-catenin, serum response factor) are not [1], [2]. These transcription factors are regulated in part by extracellular factors acting in a receptor-mediated manner. Extracellular factors orchestrating muscle regeneration are released by the injured myofibers themselves and by macrophages and lymphocytes infiltrating the injured tissue.

S100B is a member of a multigenic family of Ca^2+^-binding proteins of the EF-hand type that are only expressed in vertebrates, and exerts regulatory effects both as an intracellular factor and as an extracellular signal [3]. As an extracellular signal, S100B affects functions of several cell types via engagement of the multiligand receptor for advanced glycation end-products (RAGE), a member of the immunoglobulin superfamily [4]–[6]. S100B is expressed in skeletal muscle tissue [7], [8], is found in crushed muscle extract [9], and stimulates myoblast proliferation and inhibits myogenic differentiation [9]–[11]. However, although myoblasts express RAGE [10], S100B exerts its effects on myoblasts in vitro in a RAGE-independent manner, enhancing basic fibroblast growth factor (bFGF) receptor 1 (FGFR1) activity via binding to bFGF [9]. Interestingly, RAGE is not expressed in mature skeletal myofibers [12], becomes rapidly expressed in activated satellite cells upon muscle injury and is repressed at completion of regeneration [13] pointing to a physiological role of this receptor in muscle regeneration. When activated by its ligand, high mobility group box 1 (HMGB1) [14], [15], RAGE transduces a promyogenic and anti-proliferative and anti-tumor signal in cells of the myogenic lineage [12], [16], [17], and deletion of *Rage* results in delayed regeneration following injury, elevated satellite cell numbers, reduced myofiber numbers and myofiber hypertrophy [13]. However, S100B does bind to RAGE in myoblasts and recruits it into a RAGE/S100B/bFGF/FGFR1 tetracomplex resulting in enhancement of bFGF/FGFR1 signaling and blockade of RAGE promyogenic signaling [9].

The process of mouse skeletal muscle regeneration after injury consists of sequential steps such as satellite cell activation, which starts a few hours after injury and proceeds for 24–36 h; proliferation of activated satellite cells, which starts during day 2 post-injury and proceeds for ∼3 days; myoblast differentiation, which starts at day 3 post-injury and proceeds for ∼2 weeks; and myofiber maturation, which starts at day 5 post-injury and is complete by day 21–28 post-injury [18]. Use of myoblast cell lines and primary myoblasts proved essential for quantitative analyses of effects of the extracellular factors and receptors involved in the regulation of satellite cell activation and myoblast proliferation and differentiation. Analyses of myoblast differentiation are usually performed using confluent, i.e. high-density (HD) cells, a condition that allows the formation of a relatively large number of myotubes in 3–4 days in differentiation medium (DM). However, this in vitro condition might not reflect precisely the very beginning of the regeneration process in vivo: whereas myoblast proliferation starts soon after activation of satellite cells [18], proliferating myoblasts are intermingled with macrophages and lymphocytes whose presence might limit myoblast-myoblast contacts thus reducing local myoblast density.

We show here that injured muscle tissue releases S100B, bFGF and HMGB1 with different time courses: S100B is maximally released at day 1 post-injury, bFGF is minimally released at day 1 post-injury peaking at day 5, and HMGB1 is released starting at day 3 post-injury peaking between days 7 and 14. We also show that in low-density (LD) myoblast cultures and as long as the myoblast density is low, S100B (in the 0.1–1 nM range) interacts with and activates RAGE, but not bFGF/FGFR1 and simultaneously stimulates proliferation and activates the myogenic program; washing out S100B after 24 h of treatment results in a faster decrease in the proliferation rate and a higher level of differentiation during the next 24 h compared with S100B-untreated myoblasts. By contrast, prolonging the treatment of LD myoblasts with S100B for 3 or more days results in stimulation of proliferation and inhibition of differentiation as a result of a switch of the initial LD culture to an HD culture and consequent formation of the RAGE/S100B/bFGF/FGFR1 tetracomplex. However, raising the S100B dose to 100 nM in LD myoblast cultures results in activation of bFGF/FGFR1 and RAGE blockade, provided bFGF is present. Thus, S100B differentially activates RAGE and bFGF/FGFR1 depending on its own concentration, the presence or absence of bFGF, and myoblast density. We discuss these results in the context of the muscle regeneration process.

## Results

### S100B is Found in Conditioned Medium from Injured Skeletal Muscle Tissue

**Figure 1:**
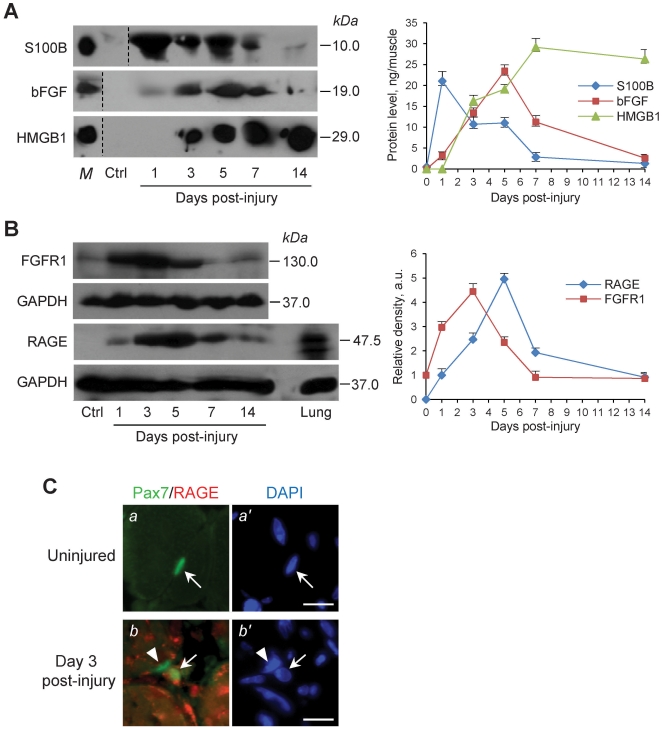
S100B, bFGF and HMGB1 are released from injured muscles. (**A**) CM from uninjured or BaCl2-injured *Tibialis anterior* muscles were processed for detection of S100B, bFGF and HMGB1 by Western blotting. S100B, bFGF and HMGB1 in CM were quantified by comparing the density of individual western blot bands in the CM with that of increasing amounts of each purified protein in parallel western blots. (**B**) After collection of CM, muscle tissue was homogenized and subjected to Western blotting for detection of RAGE and FGFR1 (20 µg protein loaded/lane). For experiments in A and B three animals/time point were used and the whole experiment was performed two times. Results in A and B are expressed as means ± SD. C, Freshly excised uninjured or BaCl_2_-injured *Tibialis anterior* muscles were processed for detection of Pax7 and RAGE by double-immunofluorescence. Nuclei were counterstained with DAPI. RAGE is not expressed in uninjured tissue. Arrow points to a quiescent satellite (Pax7^+^) cell (a,a′) and to a Pax7^+^/RAGE^+^ myoblast outside regenerating myofibers (b,b′). Arrowhead points to a Pax7^+^/RAGE^+^ myoblast within a regenerating myofiber (b,b′). Bars = 100 µm.

To have a more detailed picture of leakage of S100B from injured muscle tissue we collected the conditioned medium (CM) from muscles at varying time points after injury and measured S100B therein. We extended our analyses to released bFGF and HMGB1. We found that levels of S100B in CM from injured muscles amounted to ∼21 ng/muscle at day 1 post-injury, i.e. in coincidence with satellite cell activation, remaining relative high (i.e., ∼10 ng/muscle) during the next 4 days post-injury, i.e. during the satellite cell proliferation and differentiation phases, and declining to ∼3 ng/muscle at day 7 post-injury, i.e. during late myoblast differentiation phase, and to control values by day 14 post-injury, i.e. by the end of the myoblast differentiation process (Fig. 1A). The high level of S100B detected at day 1 post-injury likely reflected leakage of the protein from injured myofibers whereas the lower though relatively high levels found during the next 6 days might reflect ongoing clearance of leaked S100B and/or diminishing S100B leakage consequent to myofiber repair. Indeed, myofibers, quiescent satellite cells and proliferating myoblasts express S100B [7]–[9], and a subset of S100B^+^ lymphocytes [19] infiltrating the injured tissue might secrete the protein as well. By contrast, levels of bFGF amounted to ∼3 ng/muscle at day 1 post-injury, peaked (∼23 ng/muscle) at day 5 and declined to control levels between days 7 and 14 post-injury (Fig. 1A). HMGB1 was not detectable in CM at day 1 post-injury and accumulated during the next days remaining high till day 14 post-injury (Fig. 1A). Using the tissue remaining after collection of CM, we found that RAGE was not expressed in uninjured muscle tissue whereas it was expressed during the day 1 to 14 post-injury interval with a peak at day 5 (Fig. 1B), and it was localized to activated satellite (Pax7^+^) cells after injury (Fig. 1C). Also, FGFR1 was highly expressed at days 1, 3 and 5 post-injury declining thereafter (Fig. 1B). Thus, at the time of satellite cell activation injured muscle tissue released high amounts of S100B, very small amounts of bFGF, and no HMGB1, and RAGE was expressed early after injury and FGFR1 accumulated coincidently with S100B release but in advance of bFGF release (Fig. 1A,B).

Given the time-course of S100B, bFGF and HMGB1 release from injured muscles (Fig. 1A), the time-course of satellite cell proliferation and differentiation following muscle injury [18], and the transient expression of RAGE in activated, proliferating and differentiating satellite cells in injured muscles ([13]; also see Fig. 1B), we analyzed potential RAGE-mediated effects of S100B. For quantitative analyses we used C2C12 myoblasts, an established model of activated satellite cells, and primary myoblasts isolated from neonatal wild-type (WT) and *Rage* ^−/−^ mice.

### S100B Effects on Myoblast Differentiation Vary Depending on Cell Density and the Duration of Treatment

**Figure 2:**
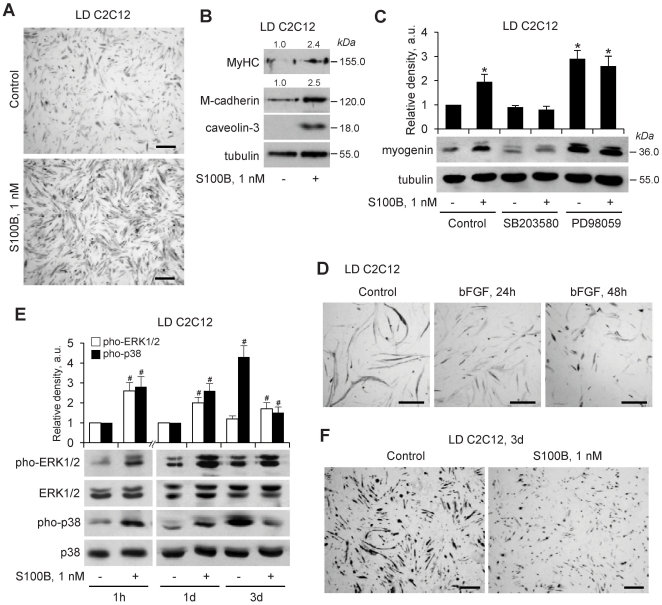
S100B effects on differentiation vary depending on myoblast density and the duration of treatment with S100B. (**A**) LD myoblasts were cultivated in DM in the absence or presence of S100B for 24 h, washed and cultivated for another 24 h in DM with no additions before MyHC immunostaining. (**B**) Same as in A except that LD myoblasts were subjected to Western blotting for detection of MyHC, M-cadherin and caveolin-3 at 24 h after washout (*n* = 3). (**C**) LD C2C12 myoblasts were cultivated for 1 h in DM with the p38 inhibitor, SB203580 (2 µM) or the MEK-ERK1/2 inhibitor, PD90059 (20 µM) and then for 24 h in the absence or presence of S100B, and subjected to Western blotting at 24 h after washout using anti-myogenin antibody (*n* = 3). (**D**) Same as in A except that LD myoblasts were treated with bFGF for 24 h and immunostained for MyHC at 24 and 48 h after bFGF washout. (**E**) LD myoblasts were cultivated in DM for 1, 24 or 72 h in the absence or presence of S100B and analyzed for ERK1/2 and p38 phosphorylation by Western blotting (*n* = 3). (**F**) LD myoblasts were cultivated in DM for 72 h in the absence or presence of S100B and subjected to MyHC immunostaining. *Significantly different from control. ^#^Significantly different from internal control. Bars = 100 µm (A,F).

As mentioned in Introduction, S100B stimulates proliferation and inhibits differentiation in HD myoblast cultures by enhancing bFGF/FGFR1 signaling [9]. To mimic the situation found early after muscle injury, i.e. when the population and/or the local density of proliferating satellite cells are relatively small, we seeded myoblasts at a low (i.e., ∼8×103 cells/cm^2^) density in growth medium (GM) for 24 h and then switched the cultures to DM for 24 h plus or minus S100B and for another 24 h to DM without S100B. At the end of this treatment, S100B-treated LD cultures showed enhanced myotube formation (Fig. 2A), increased levels of the late myogenic marker, myosin heavy chain (MyHC), and of M-cadherin and caveolin-3, that are known to be required for myoblast fusion [20]–[22] (Fig. 2B), and increased levels of the early myogenic marker, myogenin (Fig. 2C), compared with controls. Notably, when this experiment was performed using bFGF instead of S100B, inhibition of differentiation was obtained (Fig. 2D), suggesting that in LD myoblast cultures S100B might not enhance bFGF/FGFR1 signaling, i.e. that S100B and bFGF might activate different receptors. Contrary to HD myoblasts in which S100B activated ERK1/2 and inhibited p38 MAPK [9], in LD cultures S100B activated both, ERK1/2 and p38 MAPK, during the 24-h treatment (Fig. 2E), and treatment of cultures with SB203580, a specific inhibitor of the promyogenic p38 MAPK [23]–[27], inhibited myogenin expression in S100B-treated and control cultures (Fig. 2C), as expected. In contrast, PD98059, a specific inhibitor of the mitogenic MEK-ERK1/2, stimulated myogenin expression to a similar extent in S100B-treated and control LD cultures (Fig. 2C) as expected [28]. A short-term treatment of LD primary mouse myoblasts with S100B also resulted in enhanced myogenin expression and myotube formation after S100B washout (Fig. S1). Collectively, these results suggested that S100B-dependent stimulation of bFGF/FGFR1 activity might not be the mechanism underlying S100B effects in LD myoblasts. However, chronic treatment of LD myoblasts with S100B caused stimulation of ERK1/2 and inhibition of p38 MAPK (Fig. 2E) and inhibition of differentiation (Fig. 2F) similar to HD myoblasts [9].

### At Early Differentiation Stages S100B Exerts Mitogenic Effects in LD and HD Myoblasts and Activates the Myogenic Program in LD Myoblasts

**Figure 3:**
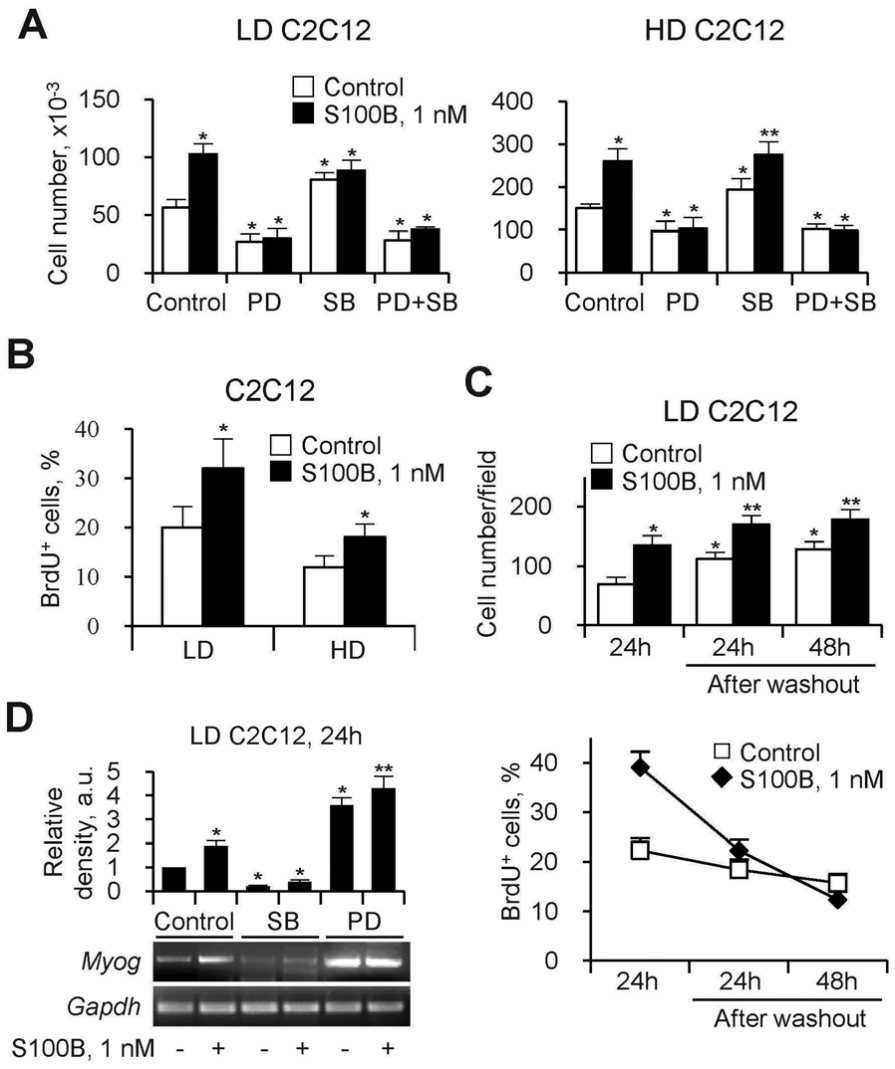
At early differentiation stages S100B exerts mitogenic effects in LD and HD myoblasts and activates the myogenic program in LD myoblasts. (**A**) LD and HD myoblasts were cultivated for 24 h in DM in the absence or presence of S100B plus or minus 20 µM PD98059, 2 µM SB203580 or both before cell counts. (**B**) Parallel cells cultivated for 24 h in DM in the absence or presence of S100B were subjected to BrdU incorporation assay. (**C**) LD myoblasts were cultivated for 24 h in DM in the absence or presence of S100B and either subjected to BrdU incorporation assay or washed and cultivated in DM for another 24 or 48 h without additions before BrdU incorporation assay. Results are expressed as cell numbers/field (top panel) and percentages of BrdU+ cells (bottom panel). (**D**) LD myoblasts cultivated for 24 h in DM in the absence or presence of S100B plus or minus 20 µM PD98059 or 2 µM SB203580 were subjected to RT-PCR to detect myogenin mRNA. *Significantly different from control. **Significantly different from control (first column from left) and internal control.

S100B increased myoblast numbers and proliferation in both, LD and HD cultures in DM (Fig. 3A,B). Smaller cell numbers were measured in LD and HD cultures treated with PD98059, compared with controls, and S100B was without effects under these conditions (Fig. 3A,B). By contrast, treatment with SB203580 increased the cell number in control cultures, and S100B further increased it in HD but not LD cultures (Fig. 3A,B). Thus, the ability of S100B to activate p38 MAPK in LD myoblasts, as opposed to the inhibitory effect of the protein on p38 MAPK in HD myoblasts [9], might serve a function other than stimulation of proliferation. In the presence of PD98059 plus SB203580, cell numbers decreased in control LD and HD cultures likely due to inhibition of MEK-ERK1/2, and S100B was without effects (Fig. 3A,B). Collectively, these results suggested that the ability of S100B to promote myoblast proliferation in LD and HD cultures in DM relied on stimulation of MEK-ERK1/2 and that the inability of S100B to stimulate proliferation in SB203580-treated LD myoblasts might be dependent on lack of S100B-dependent enhancement of bFGF/FGFR1 activity in this condition.

Cell counts of LD myoblast cultures in DM showed that S100B significantly increased cell numbers at the end of the 24-h treatment compared with controls (i.e., ∼135 cells/field vs. ∼70 cells/field) (Fig. 3C, top panel). At 24 and 48 h after S100B/vehicle washout cell numbers were higher in S100B-treated and untreated cultures compared with their respective controls; however the percent increment was larger in the case of control cultures than in S100B-treated cultures (Fig. 3C, top panel) pointing to a lower proliferation rate of S100B-treated myoblasts compared with controls after washout. Indeed, by a BrdU incorporation assay (Fig. 3C, bottom panel) of control LD cultures we found that ∼22%, 18% and 15% of the cells were in the S phase of the cell cycle at the end of the first 24-h in DM and at 24 h and 48 h after vehicle washout, respectively, while in S100B-treated cultures ∼39%, 22% and 12% of the cells were in S phase, respectively. Thus, S100B increased the fraction of proliferating myoblasts and total cell numbers during the 24-h treatment of LD cultures, and both S100B-treated and control myoblasts reduced their proliferation rate after vehicle/S100B washout. However, a larger and faster reduction of the proliferation rate occurred in S100B-treated than in control cells after washout. Also, at the end of the 24-h treatment, S100B-treated myoblasts showed a ∼90% increase in myogenin mRNA compared with controls, and inhibition of p38 MAPK resulted in similar and low myogenin mRNA levels whereas inhibition of ERK1/2 resulted in enhanced myogenin mRNA levels in S100B-treated and control myoblasts (Fig. 3D) as expected [28]. These results suggested that in LD myoblasts S100B increased the cell number via stimulation of MEK-ERK1/2 and activated the myogenic program via stimulation of p38 MAPK, and that the combination of these two events determined an enhanced differentiation after S100B washout, compared with controls.

### S100B Activates RAGE, but not bFGF/FGFR1 in LD Myoblasts

The different outcomes of short-term treatment of LD and HD myoblasts with S100B appeared to be dependent mostly on the different effect of the protein on p38 MAPK during the first 24 h of treatment: stimulation in LD cultures (Fig. 2E) and inhibition in HD cultures [9]. We reported that in HD myoblast cultures S100B promotes the formation of a RAGE/S100B/bFGF/FGFR1 tetracomplex resulting in enhancement of bFGF/FGFR1 signaling and blockade of RAGE signaling and, hence, in stimulation of ERK1/2 and proliferation and inhibition of p38 MAPK and differentiation [9].

**Figure 4:**
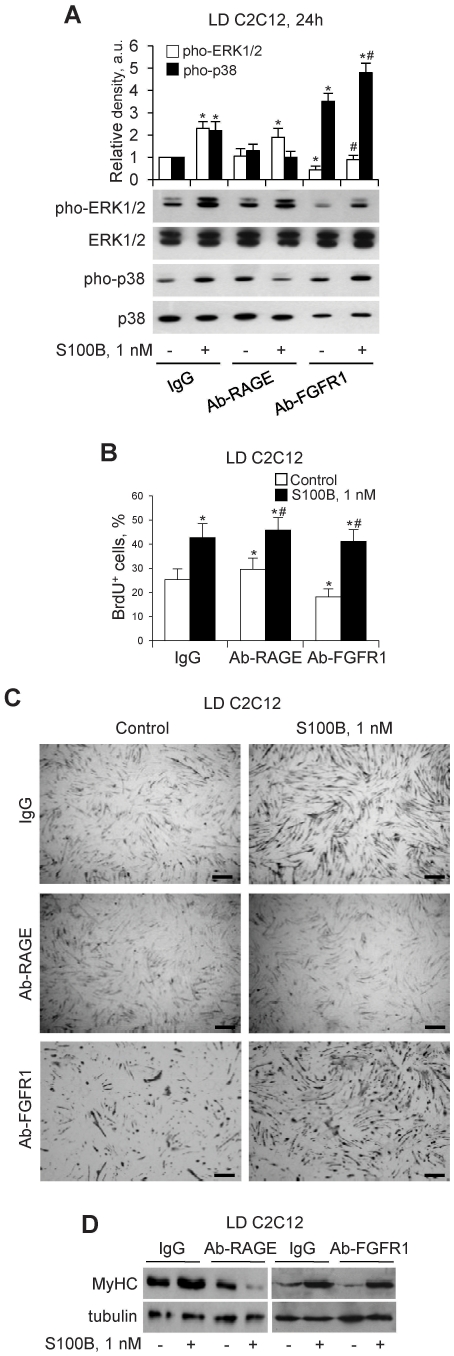
S100B activates RAGE, but not bFGF/FGFR1 in LD myoblasts. (**A**) LD myoblasts in DM were pretreated with non-immune IgG, anti-RAGE antibody or anti-FGFR1 antibody (2 h) and cultivated for 24 h in the absence or presence of S100B. Cultures were subjected to Western blotting to detect phosphorylated ERK1/2 and p38 (*n* = 3). (**B**) Same as in A except that cells pretreated with anti-RAGE or anti-FGFR1 antibody were cultivated for 24 h in the absence or presence of S100B and processed for proliferation by a BrdU incorporation assay (*n* = 3). (**C,D**) Same as in B except that cells pretreated with anti-RAGE or anti-FGFR1 antibody were cultivated for 24 h in the absence or presence of S100B, washed and cultivated in DM for another 24 h without additions and processed for MyHC immunostaining (C) or Western blotting of MyHC (D) (*n* = 3). *Significantly different from control (first column from left). **^#^**Significantly different from internal control. *n* = 3. Bars = 100 µm (C).

**Figure 5:**
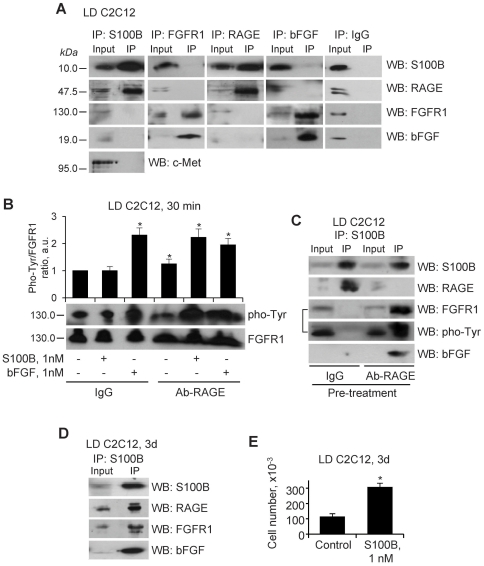
RAGE, but not bFGF/FGFR1 co-immunoprecipitates with S100B in LD myoblasts at early differentiation stages. (**A**) LD myoblasts cultivated in DM for 30 min in the presence of 1 nM S100B were subjected to immunoprecipitation using anti-S100B, anti-FGFR1, anti-bFGF or anti-RAGE antibody. The immunoprecipitates were subjected to Western blotting for detection of S100B, FGFR1, bFGF, or RAGE. In control samples non-immune IgG (IP-IgG) were used. Twenty µg protein were loaded in input lanes. (**B**) LD myoblasts were cultivated in DM for 30 min in the absence or presence of either 1 nM S100B or 1 nM bFGF after pretreatment with IgG (10 µg/ml) or anti-RAGE antibody (10 µg/ml). Cells were lysed and cell lysates were subjected to Western blotting for detection of phosphorylated Tyr and FGFR1. (**C**) LD myoblasts cultivated in DM for 2 h in the presence of either non-immune IgG (10 µg/ml) or anti-RAGE antibody (10 µg/ml). Cultures were then added with 1 nM S100B for 30 min and subjected to immunoprecipitation using anti-S100B antibody. Immunoprecipitates were probed by Western blotting with anti-S100B, anti-FGFR1, anti-bFGF or anti-RAGE antibody. The WB:FGFR1 filter was reprobed with anti- phosphorylated Tyr antibody (marked by the “[” symbol). (**D**) LD myoblasts were cultivated in DM for 3 days in the presence of 1 nM S100B without medium renovation and subjected to immunoprecipitation using anti-S100B antibody. The immunoprecipitates were probed by Western blotting with anti-S100B, anti-FGFR1, anti-bFGF or anti-RAGE antibody. (**E**) LD myoblasts were cultivated in DM for 3 days in the presence of 1 nM S100B without medium renovation and counted. *Significantly different from control.

In LD myoblasts, neutralization of FGFR1 resulted in decreased basal ERK1/2 phosphorylation levels and increased basal p38 MAPK phosphorylation levels, and S100B activated both kinases akin to native conditions (Fig. 4A). In this condition basal proliferation and differentiation were significantly reduced, and S100B stimulated proliferation and, after washout, differentiation (Fig. 4B,C). The decreased basal differentiation of anti-FGFR1-treated LD myoblasts might be due in part to the significant reduction in the myoblast number consequent to reduced proliferation (Fig. 4B), which further reduced the cell-cell contacts required for fusion into myotubes and for N-cadherin-mediated activation of the promyogenic p38 MAPK [29]. On the other hand, S100B-induced enhancement of differentiation of anti-FGFR1-treated LD myoblasts might be due to S100B ability to activate the promyogenic p38 MAPK RAGE-dependently in this condition (Fig. 4A). In the presence of anti-RAGE antibody no major changes in basal ERK1/2 and p38 MAPK phosphorylation levels compared with controls were detected, and S100B stimulated ERK1/2 and inhibited p38 MAPK (Fig. 4A), and stimulated proliferation (Fig. 4B) and inhibited differentiation (Fig. 4C,D) similar to HD myoblasts under native conditions [9]. Thus, we tentatively concluded that short-term treatment of LD myoblasts with S100B resulted in activation of RAGE, but not bFGF/FGFR1, in contrast to activation of bFGF/FGFR1 but not RAGE in HD myoblasts. However, blocking RAGE in LD cultures made S100B able to activate bFGF/FGFR1 as inferred by the S100B-dependent enhancement of proliferation and reduction of differentiation in this condition (Fig. 4B,D; also see Fig. 5B).

### RAGE, but not bFGF/FGFR1 Co-immunoprecipitates with S100B in LD Myoblasts at Early Differentiation Stages

Whereas in HD myoblast cultures RAGE and FGFR1 co-immunoprecipitated with S100B [9], in LD cultures RAGE, but not FGFR1 co-immunoprecipitated with S100B, and S100B co-immunoprecipitated with RAGE but not FGFR1 (Fig. 5A). In the absence of added S100B a certain amount of the protein was found in S100B immunoprecipitates (Fig. S2A) likely reflecting the presence of the protein in commercial sera [10], and adding S100B (1 nM) to myoblast cultures resulted in larger amounts of the protein in S100B immunoprecipitates (Fig. S2A) as expected. Also, bFGF was present in culture media of LD myoblasts cultivated for 30 min in DM in the absence or presence of added S100B (Fig. S2B). As in the case of HD myoblast cultures [9] no Met was detected in the S100B immunoprecipitate (Fig. 5A) indicating that effects of S100B on LD myoblasts were not mediated by the hepatocyte growth factor receptor, Met, either. No S100B or bFGF co-immunoprecipitated with bFGF or S100B, respectively, in LD myoblasts (Fig. 5A). Accordingly, FGFR1 was not found in S100B immunoprecipitates (Fig. 5A), S100B was not found in FGFR1 immunoprecipitates (Fig. 5A), and no S100B-dependent increase in FGFR1 phosphorylation (activation) levels could be observed (Fig. 5B) in contrast to the S100B-induced enhancement of FGFR1 phosphorylation levels in the presence of bFGF in HD myoblasts [9]. Thus, at early differentiation stages of LD myoblast cultures S100B bound to and engaged RAGE thereby simultaneously stimulating the mitogenic ERK1/2 and the promyogenic p38 MAPK: S100B/RAGE-dependent activation of ERK1/2 resulted in stimulation of proliferation whereas S100B/RAGE-dependent activation of p38 MAPK resulted in activation of the myogenic program. However, neutralizing RAGE in LD myoblasts resulted in co-immunoprecipitation of bFGF and FGFR1 with S100B (Fig. 5C) and enhancement of FGFR1 phosphorylation (Fig. 5B,C).

As a long-term treatment of LD cultures with S100B caused inhibition of differentiation (Fig. 2E), we performed immunoprecipitation analyses using LD cultures exposed to S100B for 3 days. Under this condition RAGE, bFGF and FGFR1 co-immunoprecipitated with S100B (Fig. 5D) similar to HD myoblasts [9]. This latter result was likely due to the S100B-dependent switch of the initial LD culture to an HD culture: indeed, treatment of LD cultures for 3 days with S100B resulted in a ∼3-fold increase in the cell number (Fig. 5E).

Given the multiligand nature of RAGE, we asked whether another RAGE activator, i.e. HMGB1 [14], [15], exerted the same effects in LD myoblasts as did S100B. HMGB1 was shown to exert promyogenic and anti-proliferative effects via RAGE ligation in HD myoblasts [12], [16], and to promote muscle regeneration when infused into dystrophic [30] or ischemic [31] muscle tissue. During the first 24 h of treatment HMGB1 activated ERK1/2 and p38 MAPK (Fig. S3A), stimulated proliferation (Fig. S3B) and activated the differentiation program (Fig. S3C) as did S100B. At 24 h after washout HMGB1-treated myoblasts expressed higher MyHC levels than untreated myoblasts (Fig. S3D). Also, RAGE, but not FGFR1 co-immunoprecipitated with HMGB1 (Fig. S3E), and HMGB1 did not co-immunoprecipitate with either bFGF, S100B or FGFR1 irrespective of myoblast density (unpublished data). Together, these results pointed to a similar, if not identical mechanism of action of S100B and HMGB1 in LD myoblasts while establishing a fundamental difference between these two RAGE ligands in HD myoblasts.

### At High Doses S100B Promotes the Formation of a RAGE/S100B/bFGF/FGFR1 Tetracomplex in LD Myoblasts at Early Differentiation Stages

**Figure 6:**
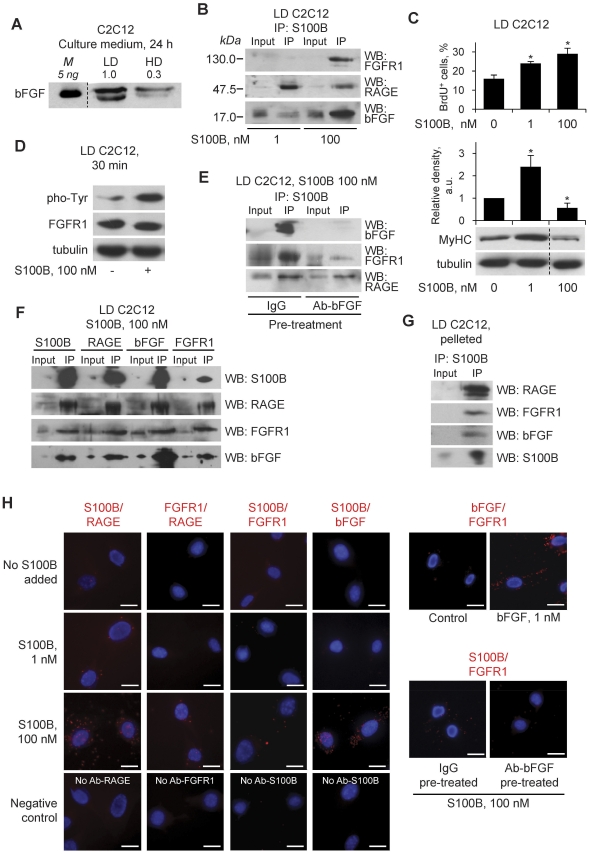
At high doses S100B promotes the formation of a RAGE/S100B/bFGF/FGFR1 tetracomplex in LD myoblasts at early differentiation stages. (**A**) HD myoblasts (one dish) and LD myoblasts (four dishes) were cultivated in DM for 24 h. Culture media were collected and subjected to Western blotting using anti-bFGF antibody. After collection of culture media the cells were lysed for Western blotting of tubulin. (**B**) LD myoblasts were cultivated in DM for 30 min in the presence of 1 or 100 nM S100B and subjected to immunoprecipitation using anti-S100B antibody. The immunoprecipitates were probed with anti-FGFR1, anti-RAGE or anti-bFGF antibody. (**C**) LD myoblasts were cultivated in DM for 24 h in the presence of varying S100B concentrations and subjected to BrdU incorporation assay to measure proliferation at the end of the 24-h treatment (upper panel). Parallel cells were washed after the first 24-h treatment, further cultivated in DM for 24 h without additions and subjected to Western blotting for detection of MyHC (lower panel). (**D**) LD myoblasts were cultivated in DM for 30 min in the absence or presence of 100 nM S100B. Cells were lysed and cell lysates were subjected to Western blotting for detection of pho-Tyr and FGFR1. (**E**) LD myoblasts were cultivated in DM for 2 h in the presence of either 40 µg/ml non-immune IgG or 40 µg/ml anti-bFGF antibody followed by addition of vehicle or S100B to 100 nM for 30 min and immunoprecipitation using anti-S100B antibody. The immunoprecipitates were probed with anti-bFGF, anti-FGFR1 or anti-RAGE antibody. (**F**) LD myoblasts cultivated in DM for 30 min in the presence of 100 nM S100B were subjected to immunoprecipitation using anti-S100B, anti-FGFR1, anti-bFGF or anti-RAGE antibody. The immunoprecipitates were subjected to Western blotting for detection of S100B, FGFR1, bFGF, or RAGE. (**G**) LD myoblasts were cultivated in GM for 24 h, washed, scraped using PBS and collected by centrifugation. The pellet was resuspended in DM (5 ml, i.e. the same volume as used during the cultivation in petri dishes) containing 1 nM S100B, centrifuged at 500× g for 5 min at room temperature and incubated at 37°C for 30 min without aspiring the supernatant. By this procedure, cell-cell contacts of the original LD culture were augmented thus artificially reproducing the conditions of an HD culture. The supernatant was aspired, and the pellet was resuspended in PBS containing 1 mM CaCl_2_ and centrifuged as above. The pellet was resuspended in 1 ml of lysis buffer for subsequent immunoprecipitation using anti-S100B antibody. The immunoprecipitate was probed with anti-RAGE, anti-FGFR1, anti-S100B or anti-bFGF antibody. (**H**) Complex formation among S100B, bFGF, FGFR1 and RAGE in LD myoblasts as investigated by in situ proximity ligation assay (PLA). LD myoblasts in DM were incubated for 30 min with varying S100B concentrations as indicated or with 1 nM bFGF. LD myoblasts in DM were also treated with either 5 µg/ml non-immune IgG or 5 µg/ml anti-bFGF antibody followed by addition of vehicle or S100B to 100 nM for 30 min. Cells were then subjected to in situ PLA using antibody pairs as indicated on top of panels. In control experiments one antibody of individual pairs was omitted as indicated. *Significantly different from control. Bars = 20 µm (H).

Next we asked why S100B was unable to activate bFGF/FGFR1 in LD myoblast cultures at early differentiation stages. At 24 h after the switch to DM, LD culture media contained ∼3-times higher bFGF levels than HD culture media (Fig. 6A) pointing to differential secretion of bFGF by C2C12 myoblasts depending on cell density. However, despite the relatively high levels of bFGF in LD culture media, no S100B-dependent stimulation of bFGF/FGFR1 signaling or S100B/bFGF/FGFR1 complex formation occurred (Figs. 5A–C). Yet, when LD cultures were treated with a 100-times higher (i.e., 100 nM vs. 1 nM) S100B dose, RAGE, bFGF and FGFR1 were detected in S100B immunoprecipitates (Fig. 6B), and stimulation of proliferation and reduction of MyHC levels were observed at 24 h after S100B washout (Fig. 6C) along with stimulation of FGFR1 phosphorylation (Fig. 6D) akin to HD cultures treated with 1 nM S100B [9]. But, when the immunoprecipitation assay was performed using LD cultures pretreated with a bFGF neutralizing antibody and exposed to 100 nM S100B, RAGE but essentially no FGFR1 or bFGF was detected in S100B immunoprecipitates (Fig. 6E). Thus, in LD myoblasts a RAGE/S100B/bFGF/FGFR1 tetracomplex formed at high S100B doses and for this to occur the presence of bFGF was required whereas in the absence of bFGF S100B invariably bound to RAGE. The simplest explanation for the different results obtained with low and high S100B in LD myoblasts was that S100B has a higher binding affinity to RAGE than to bFGF/FGFR1 and/or that the larger amount of high-order S100B oligomers forming at high S100B concentrations compared with low concentrations [32], [33] might cause S100B-dependent cross-linking of RAGE and bFGF/FGFR1. As an additional but not alternative possibility, at high doses S100B might interact separately with RAGE and bFGF/FGFR1 in LD myoblasts. We analyzed these possibilities by a combination of co-immunoprecipitation and in situ proximity ligation assay (PLA) [34].

By co-immunoprecipitation assay we found that upon treatment of LD myoblasts with 100 nM S100B all binding partners, i.e. S100B, bFGF, RAGE and FGFR1, were found in immunoprecipitates irrespective of whether anti-S100B, anti-bFGF, anti-RAGE or anti-FGFR1 antibody was used to generate the immunoprecipitates (Fig. 6F). Thus, at high doses S100B likely promoted the formation of a RAGE/S100B/bFGF/FGFR1 tetracomplex in LD myoblasts at early differentiation stages in contrast to lack of formation of such a complex at low S100 doses. These differences were likely to depend on the larger amounts of high-order S100B oligomers at 100 nM than at 1 nM [32], [33] given that all other experimental parameters were constant. By in situ PLA, S100B/RAGE complexes were detected on myoblasts when S100B was used at 1 nM, and a larger number of S100B/RAGE complexes formed when S100B was used at 100 nM (Fig. 6G). In accordance with the co-immunoprecipitation results in 5A,C,D and 6B,E, no S100B-induced close association between RAGE and FGFR1 could be detected in LD myoblasts treated with 1 nM S100B (Fig. 6H), whereas such a close association was observed in LD myoblasts treated with 100 nM S100B, provided bFGF was present (Fig. 6H). Also in accordance with the co-immunoprecipitation results in Figs. 5A and 6B, no S100B/bFGF immunocomplex formation was detected when S100B was used at 1 nM, whereas S100B/bFGF complexes formed when S100B was used at 100 nM (Fig. 6H). A relatively small number of bFGF/FGFR1 immunocomplexes were detected under basal conditions (i.e., in the absence of additions) (Fig. 6H) likely reflecting the presence of a certain amount of bFGF in the culture medium (Figs. 5A, IP:bFGF and S2B), with relatively large numbers being observed upon addition of bFGF to 1 nM to the culture as expected (Fig. 6H). Also, whereas relatively large numbers of S100B/RAGE and S100B/bFGF immunocomplexes per myoblast were detected when S100B was used at 100 nM (Fig. 6H), relatively small numbers of S100B/FGFR1 and of RAGE/FGFR1 immunocomplexes were detected in this same condition (Fig. 6H). These latter results were consistent with the notion that S100B can physically interact with RAGE and, under appropriate conditions, FGFR1-bound bFGF, but not with FGFR1 [9], and pointed to a longer distance between S100B and FGFR1 and between RAGE and FGFR1 than between S100B and RAGE and between S100B and bFGF in immunocomplexes (see [34] for a comprehensive discussion of interpretation of in situ PLA results). Together, these results suggested that the S100B binding affinity to RAGE is higher than to bFGF/FGFR1 thus promoting the formation of S100B/RAGE complexes and precluding the formation of RAGE/S100B/bFGF/FGFR1 tetracomplexes in LD myoblast culture treated with 1 nM S100B, and that at high concentrations S100B might exist in the form of oligomers capable of cross-linking RAGE and bFGF/FGFR1 in LD myoblasts (see Discussion).

We also found that a RAGE/S100B/bFGF/FGFR1 tetracomplex formed in LD cultures that had been scraped, suspended in DM containing 1 nM S100B, centrifuged (to increase the extent of cell-cell contacts), and subjected to co-immunoprecipitation using anti-S100B antibody (Fig. 6G). This result suggested that: 1) the low extent of cell-cell contacts in LD myoblasts might be one major cause of lack of formation of the RAGE/S100B/bFGF/FGFR1 complex when S100B was used at 1 nM strongly suggesting that cell density was a crucial factor determining whether low S100B activated RAGE or bFGF/FGFR1 in myoblast cultures; 2) in HD myoblast cultures low S100B promoted the formation of a RAGE/S100B/bFGF/FGFR1 transcomplex (i.e., a complex between RAGE on a cell and bFGF/FGFR1 on an apposed cell) whereas in LD myoblast cultures high S100B promoted the formation of a RAGE/S100B/bFGF/FGFR1 ciscomplex (i.e., a complex between RAGE and bFGF/FGFR1 on the same cell) (see Discussion); and 3) the increase in the myoblast number in case of chronic treatment of LD myoblasts with 1 nM S100B (Fig. 5E) created the conditions of an HD culture thus allowing the formation of the RAGE/S100B/bFGF/FGFR1 transcomplex.

### *In Vivo* Formation of S100B/RAGE and RAGE/S100B/bFGF/FGFR1 Complexes

**Figure 7:**
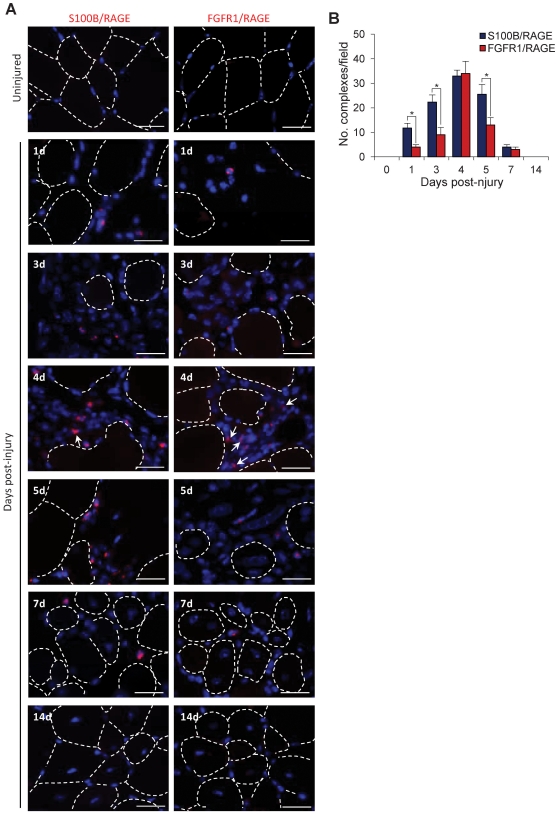
S100B/RAGE and RAGE/FGFR1 complexes in regenerating skeletal muscle tissue. (**A**) Uninjured and injured skeletal muscle tissue was subjected to in situ PLA using anti-S100B/anti-RAGE antibody pairs (left) or anti-RAGE/anti-FGFR1 antibody pairs (right). Myofiber boundaries in transverse sections are marked by dotted lines. No S100B/RAGE or RAGE/FGFR1 complexes can be detected in uninjured skeletal muscle tissue. Arrows point to RAGE/FGFR1 complexes at apposed myoblasts. RAGE/FGFR1 complexes actually represent RAGE/S100B/bFGF/FGFR1 - see text). Bars = 200 µm. (**B**) Quantitative analysis of the distribution of S100B/RAGE and RAGE/FGFR1 complexes in uninjured skeletal muscle tissue and at the indicated days post-injury. In situ complexes were counted in 10 fields (×20 magnification)/section in a total of 10 sections/time point from 2 animals. *Significantly different.

By in situ PLA, no cells exhibiting S100B/RAGE or RAGE/FGFR1 complexes could be seen in uninjured skeletal muscle tissue (Fig. 7A). However, increasing numbers of mononucleated cells exhibiting S100B/RAGE complexes were observed in skeletal muscle tissue at day 1 to day 5 post-injury in coincidence with relatively high and low S100B and bFGF levels, respectively, in CM and high RAGE levels in tissue, and decreasing numbers at days 7 and 14 post-injury in coincidence with very low S100B levels in CM and still high RAGE levels in tissue (Fig. 1A,B). Mononucleated cells exhibiting RAGE/FGFR1 complexes indicative of RAGE/S100B/bFGF/FGFR1 tetracomplex formation were seen in very low numbers at day 1 post-injury and increasing numbers at days 3 and 5 post-injury (Fig. 7A). Notably, similar numbers of mononucleated cells exhibiting S100B/RAGE and RAGE/FGFR1 complexes were observed at day 4 post-injury (Fig. 7B). Interestingly, at day 4 post-injury, i.e. when the density of mononucleated cells between myofibers was relatively high, RAGE/FGFR1 complexes were detected at contact sites of apposed cells (Fig. 7A, day 4, arrows) besides on single cells, whereas S100B/RAGE complexes were mostly seen on single cells. These results supported the possibility that S100B preferentially activates RAGE early after muscle injury (i.e., during the activation/proliferation phase) and enhances bFGF/FGFR1 signaling during the myoblast proliferation phase in vivo. However, it is possible that at single time-points during muscle regeneration, there are sites where S100B-activated RAGE signaling prevails over S100B-enhanced bFGF/FGFR1 signaling and sites where the opposite occurs.

### Low S100B Upregulates RAGE, but not FGFR1 Expression in LD Myoblasts

We recently found [13] that: 1) RAGE is sharply expressed in activated satellite cells following injury, is detected in Pax7^+^/myogenin^−^ (i.e., proliferating) satellite cells (also see Fig. 1C) and in Pax7^−^/myogenin^+^ (i.e., differentiating) satellite cells during the proliferation and differentiation phases, and is repressed at completion of regeneration; 2) maximum RAGE expression levels in injured muscle tissue are measured around day 5 post-injury (also see Fig. 1B), i.e. in coincidence with decreasing proliferation of activated satellite cells and increasing differentiation of myoblasts [18]; and 3) regeneration of injured *Rage* ^−/−^ muscles is delayed at least one week compared with wild-type muscles. Collectively, these results suggest that RAGE signaling plays a physiological role during regeneration of injured skeletal muscles. Given the time-course of S100B release from injured muscles (Fig. 1A) and considering that RAGE ligands upregulate RAGE expression [4], [5], we analyzed the possibility that S100B might contribute to increase RAGE expression in LD myoblasts.

Indeed, S100B in the 1–10 nM range increased RAGE, but not FGFR1 levels in LD myoblast cultures (Fig. S4A). However, when added to 100 nM to LD myoblast cultures, S100B enhanced FGFR1 levels while losing the ability to increase RAGE levels (Fig. S4B). Low S100B upregulated RAGE expression in LD cultures in a RAGE-dependent manner as no such effect occurred in LD cultures pretreated with a RAGE neutralizing antibody (Fig. S4C). Significantly, low S100B upregulated FGFR1 levels in LD cultures pretreated with a RAGE neutralizing antibody (Fig. S4C) likely due to the ability of S100B to enhance bFGF-FGFR1 signaling under these conditions (see Figs. 4B,C and 5B,C).

### S100B Engages RAGE in LD Primary Wild-type Myoblasts and Activates FGFR1 in LD *Rage* ^−/−^ Myoblasts

**Figure 8:**
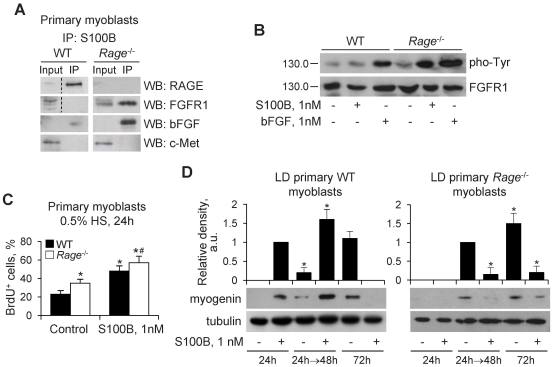
S100B engages RAGE in LD wild-type myoblasts and activates FGFR1 in *Rage* ^−/−^ myoblasts. (**A**) Primary myoblasts from WT and *Rage* ^−/−^ mice in DM were treated for 30 min with 1 nM S100B and subjected to immunoprecipitation using an anti-S100B antibody. S100B immunoprecipitates were analyzed for RAGE, FGFR1, bFGF and c-Met levels. (**B**) LD WT and *Rage* ^−/−^ myoblasts were cultivated in DM for 30 min in the absence or presence of either 1 nM S100B or 1 nM bFGF. Cells were lysed and cell lysates were subjected to Western blotting for detection of phosphorylated Tyr and FGFR1. (**C**) WT and *Rage* ^−/−^ myoblasts cultivated in DM for 24 h in the absence or presence of 1 nM S100B were subjected to BrdU incorporation assay. (**D**) WT and *Rage* ^−/−^ myoblasts were cultivated in DM in the absence or presence of 1 nM S100B for 24 or 72 h without medium renewal. One set of cells was washed and further cultivated for 48 h in DM without additions. At the end of treatments, cells were lysed and cell lysates were subjected to Western blotting to detect myogenin (as a myogenic differentiation marker). *Significantly different from control (first column from left). ^#^Significantly different from internal control (second column from left).

S100B stimulated proliferation and inhibited differentiation of HD WT and *Rage* ^−/−^ myoblasts via complex formation with bFGF/FGFR1 [9]. Whereas in LD WT myoblasts RAGE, but not FGFR1 or bFGF co-immunoprecipitated with low S100B (Fig. 8A), in the case of LD *Rage* ^−/−^ myoblasts FGFR1 and bFGF co-immunoprecipitated with low S100B (Fig. 8A) and S100B enhanced FGFR1 phosphorylation (Fig. 8B) similar to LD C2C12 myoblasts treated with anti-RAGE antibody (Fig. 5B,C). Consistently, S100B enhanced the proliferation and was unable to activate the differentiation program in LD *Rage* ^−/−^ myoblasts (Fig. 8C). Also, clearance of S100B after 24 h resulted in stimulation of myogenin expression in WT myoblasts, whereas reduction of myogenin levels was observed in *Rage* ^−/−^ myoblasts (Fig. 8D). Lastly, chronic treatment of WT and *Rage* ^−/−^ myoblasts with S100B resulted in inhibition of myogenin expression (Fig. 8D). Thus, absence of RAGE made S100B available for binding to bFGF/FGFR1 irrespective of the cell density (also see [9]), again suggesting that a higher binding affinity of S100B to RAGE than to bFGF/FGFR1 might play an important role in S100B effects in LD WT myoblast cultures.

## Discussion

We have shown that the danger signal S100B protein is released in high abundance from injured skeletal muscle tissue at day 1 post-injury, and levels of released S100B remain relatively high during the next 4 days, declining around day 7 post-injury and diminishing to control values by day 14. Thus, relatively high levels of extracellular S100B are found in injured muscles during the satellite cell activation and proliferation phases followed by a slow decrease during the myoblast differentiation phase and no obvious release during the myofiber maturation phase. The time-course of S100B release upon muscle injury and levels of released S100B point to regulatory effects of the protein during the satellite cell activation, proliferation and early differentiation phases, with the potential of activating bFGF/FGFR1 [9] or RAGE (the present study) depending on local S100B and bFGF concentrations and myoblast density and on levels of RAGE expression. Importantly, however, very low levels of bFGF are found in CM from injured muscles early after injury (Fig. 1A), whereas RAGE becomes rapidly expressed in activated satellite cells (Fig. 1C). Thus, during the first few days post-injury S100B might activate RAGE irrespective of S100B concentration and myoblast density given the very small release of bFGF, and activate bFGF/FGFR1 thereafter in coincidence with maximum release of bFGF, high FGFR1 levels and a higher myoblast density.

Using C2C12 and primary myoblasts as a model we have shown that in LD myoblast cultures low S100B engages RAGE thereby simultaneously stimulating proliferation via MEK-ERK/2 and activating the myogenic program via p38 MAPK. These results are at variance with those obtained using HD myoblast cultures in which low S100B stimulates bFGF/FGFR1 signaling and inactivates RAGE via the formation of a RAGE/S100B/bFGF/FGFR1 tetracomplex thereby enhancing myoblast proliferation and inhibiting myogenic differentiation [9]. The aforementioned S100B effects on LD myoblasts are restricted to the first 24 h of treatment in DM and result in different outcomes beyond this time interval depending on whether or not S100B persists: clearance of S100B results in a larger and faster decrease in the proliferation rate and in an enhanced myotube formation compared with controls consequent to S100B-induced increase in myoblast density and activation of the myogenic program, whereas maintaining S100B in the culture medium results in stimulation of proliferation and inhibition of differentiation as a consequence of S100B-dependent switch of the initial LD culture to an HD culture.

In LD cultures and as long as the myoblast density is low, low S100B cannot bind to bFGF/FGFR1 thus precluding the enhancement of FGFR1 signaling; S100B binding to RAGE in this condition causes stimulation of proliferation via ERK1/2 and upregulation of myogenin mRNA expression via p38 MAPK that translate into enhanced myogenic differentiation once S100B has been cleared. However, high S100B can promote the formation of a RAGE/S100B/bFGF/FGFR1 tetracomplex in LD cultures resulting in stimulation of proliferation and inhibition of differentiation. As the RAGE and FGFR1 abundance on a per cell basis at the time of switch from GM to DM was the same in LD and HD myoblast cultures (unpublished data), the inability of low S100B to interact with bFGF/FGFR1 in LD myoblasts is likely to depend on a higher binding affinity of the protein to RAGE than to bFGF/FGFR1. This conclusion is supported by the finding that, as investigated by co-immunoprecipitation assay and in situ PLA, increasing the S100B dose to 100 nM makes S100B capable of recruiting RAGE and bFGF/FGFR1 into a complex in LD in myoblasts at early differentiation stages resulting in stimulation of MEK-ERK1/2 and proliferation and inhibition of p38 MAPK and differentiation. Results obtained using C2C12 myoblasts pretreated with a RAGE neutralizing antibody and *Rage* ^−/−^ myoblasts are consistent with this conclusion: in the absence of functional RAGE, S100B interacts with bFGF/FGFR1 irrespective of myoblast density thereby enhancing the mitogenic and anti-myogenic bFGF/FGFR1 signaling. However, for this to occur the presence of bFGF is required: in the absence of bFGF S100B activates the promyogenic RAGE irrespective of the S100B concentration and myoblast density (also see [9]).

Short-term treatment of LD C2C12 myoblasts with S100B results in activation of ERK1/2 and p38 MAPK. Simultaneous activation of ERK1/2 and p38 MAPK in myoblasts in DM is counterintuitive given the established role of ERK1/2 in stimulation of proliferation [29], [35], [36] and of p38 MAPK in activation of the myogenic program [23]–[27]. The proposed role of p38 MAPK as a molecular switch from proliferation to differentiation in myoblasts [37] cannot be invoked in the present case because we performed our experiments in DM and because inhibition of p38 MAPK resulted in increased cell numbers and inhibition of differentiation. Thus, the ability of S100B to cause those apparently contrasting effects at early stages of differentiation of LD myoblasts relies on the engagement of RAGE.

**Figure 9:**
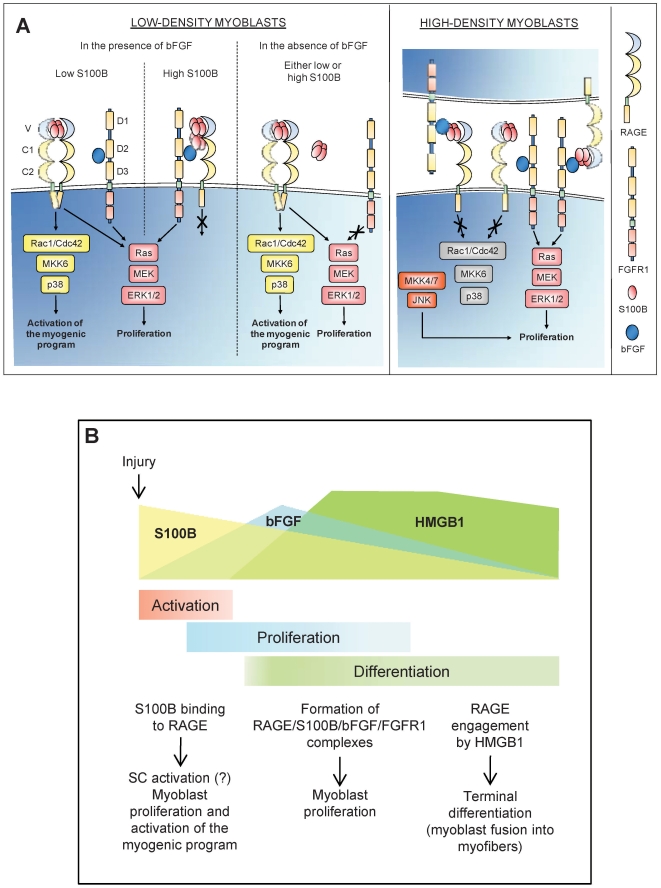
Proposed schematics of S100B/RAGE and RAGE/S100B/bFGF/FGFR1 interactions in LD and HD myoblasts and of action of released S100B following skeletal muscle injury. (**A**) (Left) In LD myoblast cultures S100B tetramers/octamers promote RAGE oligomerization and/or stabilization of RAGE oligomers resulting in the simultaneous stimulation of proliferation and activation of the myogenic program; this occurs irrespective of the absence or presence of bFGF. At high concentrations, high-order S100B oligomers bind bFGF and the S100B/bFGF complex cross-links RAGE and FGFR1 on the same cell. This results in enhancement of FGFR1 signaling and blockade of RAGE signaling likely due to lack of RAGE oligomerization and/or stabilization of RAGE oligomers. However, in the absence of bFGF either low or high S100B engages RAGE. (Right) In HD myoblast cultures, S100B tetramers/octamers bind bFGF and the S100B/bFGF complex cross-links RAGE and FGFR1 on apposed cells resulting in blockade of RAGE signaling likely due to lack of RAGE oligomerization and/or stabilization of RAGE oligomers and enhancement of FGFR1 signaling. This results in stimulation of proliferation and inhibition of differentiation. (**B**) Early after injury S100B mainly engages RAGE on activated satellite cells thereby stimulating their proliferation and activating the myogenic program. Whether S100B activates satellite cells remains to be investigated (?). During the proliferation phase S100B binds to both, RAGE and bFGF/FGFR1 and promotes the formation of a RAGE/S100B/bFGF/FGFR1 tetracomplex thereby stimulating myoblast proliferation and blocking RAGE [9]. At this stage HMGB1 might compete with S100B for binding to RAGE thereby unlocking RAGE and stimulating its promyogenic activity [12]. Later on, with diminishing S100B levels RAGE would be activated by HMGB1 thereby promoting myoblast differentiation and myocyte fusion into myofibers.

In the extracellular space, S100B forms oligomers [32], [33], and S100B causes RAGE oligomerization via binding to RAGE V domain [33] and/or stabilizes RAGE oligomers [38], which is required for RAGE signaling [39]. Based on the present results, we propose that in LD cultures treated with low S100B, S100B tetramers/octamers promote RAGE oligomerization and/or stabilize RAGE oligomers resulting in RAGE activation (Fig. 9A, left half, low S100B). By contrast, at high S100B concentrations higher-order S100B oligomers might additionally interact with FGFR1-bound bFGF resulting in the formation of a RAGE/S100B/bFGF/FGFR1 on the same cell (Fig. 9A, left half, high S100B). In this case, RAGE cannot efficiently oligomerize and, hence, cannot signal, while bFGF-FGFR1 signaling is enhanced. However, in the absence of bFGF either low or high S100B activates RAGE (Fig. 8A, left half). By contrast, in HD cultures treated with low S100B, S100B tetramers/octamers might cross-link RAGE and FGFR1-bound bFGF on apposed cells (Fig. 9A, right half) with resultant inability of non-stabilized RAGE oligomers to activate p38 MAPK and enhancement of bFGF-FGFR1 signaling.

RAGE activation by HMGB1 in HD myoblasts results in p38 MAPK-dependent stimulation of differentiation and inhibition of proliferation via p38 MAPK-dependent inhibition of ERK1/2 [12], [16]. By contrast, RAGE activation by HMGB1 in LD cultures results in activation of both p38 MAPK and ERK1/2 at early differentiation stages stimulating proliferation and activating the myogenic program similar to S100B. However, with increasing myoblast density HMGB1 stimulates differentiation and inhibits proliferation [12], [16]. Thus, HMGB1-induced ability of RAGE to stimulate differentiation and inhibit proliferation in HD cultures, as opposed to the inability of S100B to stimulate RAGE in this condition, likely reflects the ability of S100B, but not HMGB1 to bind bFGF/FGFR1. It is tempting to speculate that HMGB1-induced activation of RAGE during the first 24 h in LD myoblasts results in an extent of p38 MAPK activation sufficient to activate the myogenic program but insufficient to inhibit ERK1/2. In contrast, in HD myoblasts a more intense and sustained HMGB1/RAGE-dependent activation of p38 MAPK might result in inhibition of ERK1/2 [16]. Also, signals triggered by cell-cell contacts in HD cultures might enhance p38 MAPK activity. For example, in HD myoblast cultures N-cadherin signaling [29] and activation of the immunoglobulin superfamily member, CDO [40], activate p38 MAPK and the myogenic program, and these effects might add to HMGB1/RAGE effects.

RAGE expression in skeletal muscle tissue is developmentally regulated, RAGE being expressed up to a few postnatal days and repressed thereafter [12]. RAGE becomes transiently re-expressed in adult skeletal muscle satellite cells upon injury (Fig. 1B,C) followed by repression of RAGE expression at completion of the regeneration process, and RAGE expression and signaling in activated satellite cells are required for optimal muscle regeneration. Indeed, muscle regeneration is delayed at least one week in *Rage* ^−/−^ mice, RAGE signaling via p38 MAPK playing an important role in the induction of myogenin and myogenin-dependent repression of Pax7 expression [13]. In this context, S100B released from injured myofibers might stimulate RAGE signaling and even enhance RAGE expression in activated satellite cells (Fig. 9B), thereby stimulating myoblast proliferation and activating the myogenic program. This is a likely possibility early after injury given the sharp release of S100B from injured myofibers, the very small extent of bFGF release at day 1 post-injury (Fig. 1A) and the rapid expression of RAGE in activated satellite cells (Fig. 1B,C). However, during the proliferation phase of the regeneration process [18] when both S100B and bFGF are present extracellularly (Fig. 1A), S100B would promote the expansion of the myoblast population by enhancing bFGF/FGFR1 signaling (Fig. 8B): at this stage RAGE signaling would be reduced owing to its recruitment into a RAGE/S100B/bFGF/FGFR1 tetracomplex [9]. Yet, extracellular HMGB1, which also accumulates in the extracellular space during the proliferation phase and persists there longer than S00B and bFGF, might compete with S100B for binding to RAGE [12] thereby unlocking RAGE and enhancing RAGE promyogenic and anti-proliferative signaling (Fig. 9B). Thus, S100B and HMGB1, which both are damage-associated molecular pattern factors [3], [14], [15], might physiologically regulate the regeneration of injured muscle tissue with different mechanisms depending on their respective local concentration and myoblast density. Whether S100B also activates satellite cells in injured skeletal muscles remains to be investigated.

## Materials and Methods

### Expression and Purification of S100B

Recombinant bovine S100B, that is 97% identical to mouse S100B, was expressed and purified as reported [41], [42]. The S100B concentration was calculated using the molecular mass of the S100B dimer (21 kDa).

### Preparation of Conditioned Media from Injured Skeletal Muscle Tissue

Injury of C57BL/6 (Charles River) mouse muscles was performed by BaCl_2_ injection [43] in *Tibialis anterior* muscle of 8-wk old mice, under zolazepam/tiletamine anesthesia. Briefly, 50 µl of an aqueous 1.2% (w/v) BaCl_2_ solution were injected along the whole length of left *Tibialis anterior* muscles. Controlateral muscles were injected with vehicle and used as controls. After different recovery times (1–14 days) the animals were sacrificed and *Tibialis anterior* muscles were removed and incubated in phosphate-buffered saline (0.15 ml/muscle) for 2 h at 4°C under agitation [44]. The conditioned media (CM) so obtained were trichloroacetic acid-precipitated [45] for detection of S100B, bFGF and HMGB1 by Western blotting. After CM collection, muscle tissue was homogenized in Laemmli buffer (0.4 ml/muscle) and subjected to Western blotting to detect RAGE and FGFR1. See Text S1 for double Pax7/RAGE immunofluorescence. Approval of use of animals was obtained by the Ethics Committee of the Perugia University and the Ministero della Salute, Italy.

### Cell Culture Conditions

C2C12 and primary myoblasts seeded at a low density (LD, ∼8×10^3^ cells/cm^2^) or high density (HD, ∼32×10^3^ cells/cm^2^), were cultured for 24 h in Dulbecco's modified Eagle medium (Invitrogen) supplemented with 20% FBS (Invitrogen), 100 U/ml penicillin, and 100 µg/ml streptomycin (GM), in a H_2_O-saturated 5% CO_2_ atmosphere at 37°C and then shifted to Dulbecco's modified Eagle medium containing 0.5% horse serum (DM) to induce myoblast differentiation. C2C12 and primary myoblasts were cultivated in DM in the absence or presence of either S100B, bFGF (Peprotech) or HMGB1 plus or minus other additions as indicated in figure legends. Details of myoblast treatment after 24 h in DM are given in legends to pertinent figures.

### Isolation of Primary Myoblasts

*Rage* ^−/−^ mice generated as described [46] were obtained from Angelika Bierhaus (Heidelberg, Germany). Primary myoblasts were isolated from 3-d-old WT C57BL/6 or *Rage* ^−/−^ pups, cultivated as described [47] and characterized by immunofluorescence using a polyclonal anti-Met antibody (1∶50, Santa Cruz Biotechnology) after fixation with cold methanol for 7 min at −20°C. Greater than 95% cells were Met-positive.

### Cell Proliferation

Cell proliferation was measured by a bromodeoxyuridine (BrdU) incorporation assay. BrdU was added to cultures 2 h before fixation with cold methanol at −20°C and processing by immunofluorescence using a monoclonal anti-BrdU antibody (1∶50, Santa Cruz Biotechnology). BrdU^+^ and total cells were counted.

### Western Blotting

Myoblasts were lysed and cell lysates were subjected to Western blotting as described [10] using the antibodies listed in Text S1. Where required, culture media were collected and trichloroacetic acid-precipitated [45]. The pellets were resuspended in Laemmli buffer and subjected to Western blotting using anti-bFGF antibody. The immune reaction was developed by enhanced chemiluminescence (SuperSignal West Femto Maximum or SuperSignal West Pico, both from Pierce).

### Immunocytochemistry

MyHC was detected by immunocytochemistry as described [10].

### Reverse Transcription-PCR

Total RNA was extracted from C2C12 myoblasts using the TRIzol reagent (Invitrogen) according to the manufacturer's instructions. The following primers were used: murine myogenin 5′-GCTGTATGAAACATCCCCCTA-3′ and 5′-CGCTGTGGGAGTTGCATT-3′ (denaturation at 94°C for 1 min, annealing at 56°C for 30 sec, extension at 72°C for 30 sec).

### Co-immunoprecipitation

C2C12 and primary myoblasts cultivated for 30 min in DM in the absence or presence of S100B were lysed in buffer containing 50 mM Tris-HCl (pH 7.4), 150 mM NaCl, 1% Triton X-100, 1 mM CaCl_2_, 10 mM NaF and 1 mM sodium orthovanadate in the presence of a mixture of protease inhibitors (Roche Applied Science). Solubilized proteins were subjected to immunoprecipitation using either a polyclonal anti-S100B antibody (SWant, 2 µg/mg total protein), a polyclonal anti-RAGE antibody (Santa Cruz Biotechnology, 2 µg/mg total protein), a monoclonal anti-FGFR1 antibody (Chemicon, 2 µg/mg total protein), a polyclonal anti-bFGF antibody (Santa Cruz Biotechnology, 2 µg/mg total protein) or non-immune IgG. Immunoprecipitates were probed with the following antibodies: polyclonal anti-S100B antibody (1∶1000, Epitomics), polyclonal anti-RAGE antibody (1∶1000, Santa Cruz Biotechnology), monoclonal anti-FGFR1 antibody (1∶1000, Chemicon), polyclonal anti-bFGF antibody (1∶1000, Santa Cruz Biotechnology), polyclonal anti-c-Met antibody (1∶500, Santa Cruz Biotechnology).

### Neutralization of Ligands or Receptors

Myoblasts in DM were treated for 2 h with either polyclonal anti-RAGE antibody (10 µg/ml, Santa Cruz Biotechnology) or monoclonal anti-FGFR1 antibody (2 µg/ml, Chemicon) and cultivated for 24 h in the absence of additions or presence of S100B. In experiments of effects of neutralization of culture medium bFGF, monoclonal anti-bFGF antibody (5 µg/ml, clone bFM-1, Millipore) was added to myoblasts in DM for 2 h followed by treatments as described in figure legends. Control samples received non-immune IgG.

### *In situ* Proximity Ligation Assay

To investigate whether S100B interacts with bFGF, FGFR1 and/or RAGE we also used in situ proximity ligation assay (PLA), which allows the visualization of subcellular localization and protein-protein interactions in situ [34]. See Text S1 for details.

### Statistical analysis

Each experiment was repeated at least three times. Representative experiments are shown unless stated otherwise. The data were subjected to analysis of variance (ANOVA) with SNK post hoc analysis using a statistical software package (GraphPad Prism version 4.00, GraphPad Software, San Diego, CA, www.graphpad.com). Where appropriate data were represented as averages of 3 determinations ± SEM. Statistical significance was assumed when p<0.05.

## Supporting Information

Figure S1 Short-term treatment of LD mouse primary myoblasts with S100B results in enhanced myogenin expression and myotube formation after S100B washout. (**A**) LD primary myoblasts were cultivated in DM in the absence or presence of S100B for 24 h, washed and cultivated for another 24 h in DM with no additions. Myoblasts were lysed and cell lysates were subjected to Western blotting for detection of myogenin. (**B**) Same as in *A* except that LD myoblasts were viewed by phase-contrast at 48 h after washout. Bars = 250 µm (100 µm in insets).
(TIF)

Figure S2 Detection of S100B and bFGF in culture media. (**A**) Immunoprecipitation of S100B in LD myoblast cultures in the absence or presence of added S100B. C2C12 myoblasts cultivated for 30 min in DM in the absence (C) or presence (S100B) of 1 nM S100B were lysed in buffer containing 50 mM Tris-HCl (pH 7.4), 150 mM NaCl, 1% Triton X-100, 1 mM CaCl2, 10 mM NaF and 1 mM sodium orthovanadate in the presence of a mixture of protease inhibitors (Roche Applied Science). Solubilized proteins were subjected to immunoprecipitation using a polyclonal anti-S100B antibody (SWant, 2 µg/mg total protein). Inputs (In) and immunoprecipitates (IP) (20 µg protein each) were subjected to Western blotting using an anti-S100B antibody. (**B**) bFGF is found in culture media of LD myoblasts. C2C12 myoblasts were cultivated for 30 min in DM with no additions (control, C) or in the presence of added S100B (1 nM), and culture media were collected and trichloroacetic acid-precipitated. The pellets were resuspended in Laemmli buffer and subjected to Western blotting using anti-bFGF antibody. Purified bFGF (10 ng) was loaded on lane M.
(TIF)

Figure S3 Short-term treatment of LD C2C12 myoblasts with HMGB1 results in stimulation of proliferation and activation of the myogenic program. Myoblasts were cultivated in DM in the absence or presence of HMGB1 for 24 h, washed and cultivated for another 24 h in DM with no additions. (**A–C**) During the first 24 h of treatment HMGB1 activated ERK1/2 and p38 MAPK (*A*), stimulated proliferation (*B*) and activated the differentiation program (*C*). (**D**) At 48 h after washout HMGB1-treated myoblasts expressed higher MyHC levels than untreated myoblasts. (**E**) Myoblasts were cultivated in DM in the presence of HMGB1 for 30 min and processed for immunoprecipitation using an anti-HMGB1 antibody. RAGE, but not FGFR1 co-immunoprecipitated with HMGB1.
(TIF)

Figure S4 S100B upregulates either RAGE or FGFR1 expression in LD myoblasts depending on the concentration. (**A**) LD myoblasts were cultivated in DM for 24 h in the presence of increasing S100B concentrations and subjected to Western blotting using anti-FGFR1 or anti-RAGE antibody. (**B**) Myoblasts pretreated (2 h) with 10 µg/ml anti-RAGE antibody or 2 µg/ml anti-FGFR1 antibody were cultivated in DM for 24 h in the absence or presence of 1 nM S100B and subjected to Western blotting using anti-RAGE antibody or anti-FGFR1. Controls received 10 µg/ml non-immune IgG. *Significantly different from control (n = 3).
(TIF)

Text S1 Supplementary materials and methods. Double Pax7/RAGE Immunofluorescence. Tibialis anterior muscles were removed from uninjured mice and from injured mice at day 3 after injection with BaCl_2_, fixed in 4% formalin in tris-buffered saline (TBS) (pH 7.2), dehydrated and paraffin embedded. Paraffin sections were cut at 4 µm, deparaffinized with xylene, rehydrated in a graded ethanol series. Sections were washed with TBS, pH 7.4, incubated for 1 h with Blocking Buffer (BB, 0.4% Triton-X-100, 10% donkey serum and 1% bovine serum albumin in phosphate-buffered saline) and then probed with the following primary antibodies in BB (1∶20): goat polyclonal anti-RAGE (Santa Cruz Biotechnology), mouse monoclonal anti-Pax7 (R&D Systems). The secondary antibodies were donkey anti-mouse Alexa Fluor 488-conjugated (Invitrogen), donkey anti-goat Alexa Flour 594-conjugated (Invitrogen), and goat anti-rabbit rhodamine-conjugated (Sigma Aldrich). Western Blotting. Myoblasts were lysed and cell lysates were subjected to Western blotting as described (10). The following antibodies were used: monoclonal anti-MyHC (1∶1000, Novocastra), monoclonal anti-M-cadherin (1∶1000, Santa Cruz Biotechnology), polyclonal anti-caveolin-3 (1∶1000, BD Transduction Laboratories), monoclonal anti-α-tubulin (1∶10000, Sigma), polyclonal anti-phosphorylated (Thr180/Tyr182) p38 MAPK (1∶1000, Cell Signaling Technology), polyclonal anti-p38 MAPK (1∶2000, Cell Signaling Technology), polyclonal anti-phosphorylated (Thr202/Tyr204) ERK1/2 (1∶2000, Cell Signaling Technology), polyclonal anti-ERK1/2 (1∶20000, Sigma), monoclonal anti-myogenin (1∶1000, Santa Cruz Biotechnology), anti-S100B (1∶1000, Epitomics), polyclonal anti-RAGE antibody (1∶1000, Santa Cruz Biotechnology), monoclonal anti-FGFR1 antibody (1∶1000, Chemicon), polyclonal anti-bFGF antibody (1∶1000, Santa Cruz Biotechnology), polyclonal anti-c-Met antibody (1∶500, Santa Cruz Biotechnology), and monoclonal anti-p-Tyr (PY20) sc-508 (1∶1000, Santa Cruz Biotechnology). For analyses of culture medium bFGF, culture media were collected processed as described [17]. Pellets were resuspended in Laemmli buffer and subjected to Western blotting using anti-bFGF or anti-HMGB1 antibody (1∶1000, R&D Systems). In Situ Proximity Ligation Assay. Myoblasts in differentiation medium were pretreated with polyclonal anti-S100B (50 µg/ml, SWant), monoclonal anti-bFGF antibody (5 µg/ml, clone bFM-1, Millipore) or non-immune IgG (50 µg/ml) for 2 h before incubation with 1 nM S100B or vehicle for 30 min. Cells were then fixed as described [10], treated with a mixture of either anti-RAGE and anti-FGFR1 antibodies, anti-S100B and anti-bFGF antibodies, anti-S100B and anti-FGFR1 antibodies or anti-S100B and anti-RAGE antibodies, and subjected to in situ PLA [34] (OLINK Bioscience, Uppsala) according to the manufacturer's instructions. In control experiments anti-RAGE antibody, anti-FGFR1 antibody, anti-S100B antibody or anti-bFGF antibody was omitted. Skeletal muscle tissue from uninjured and injured mice was subjected to in situ PLA using anti-S100B/anti-RAGE antibody pairs or anti-RAGE/anti-FGFR1 antibody pairs. Complex formation between RAGE and FGFR1 was taken as representative of RAGE/S100B/bFGF/FGFR1 complex formation (see [9] and Fig. 6H).
(TIF)
