# Supplementary figures and images for: S100B Engages RAGE or bFGF/FGFR1 in Myoblasts Depending on Its Own Concentration and Myoblast Density. Implications for Muscle Regeneration
Source: PLoS One. 2012 Jan 20;7(1):e28700. doi: 10.1371/journal.pone.0028700 (PMC3262793; doi:10.1371/journal.pone.0028700)

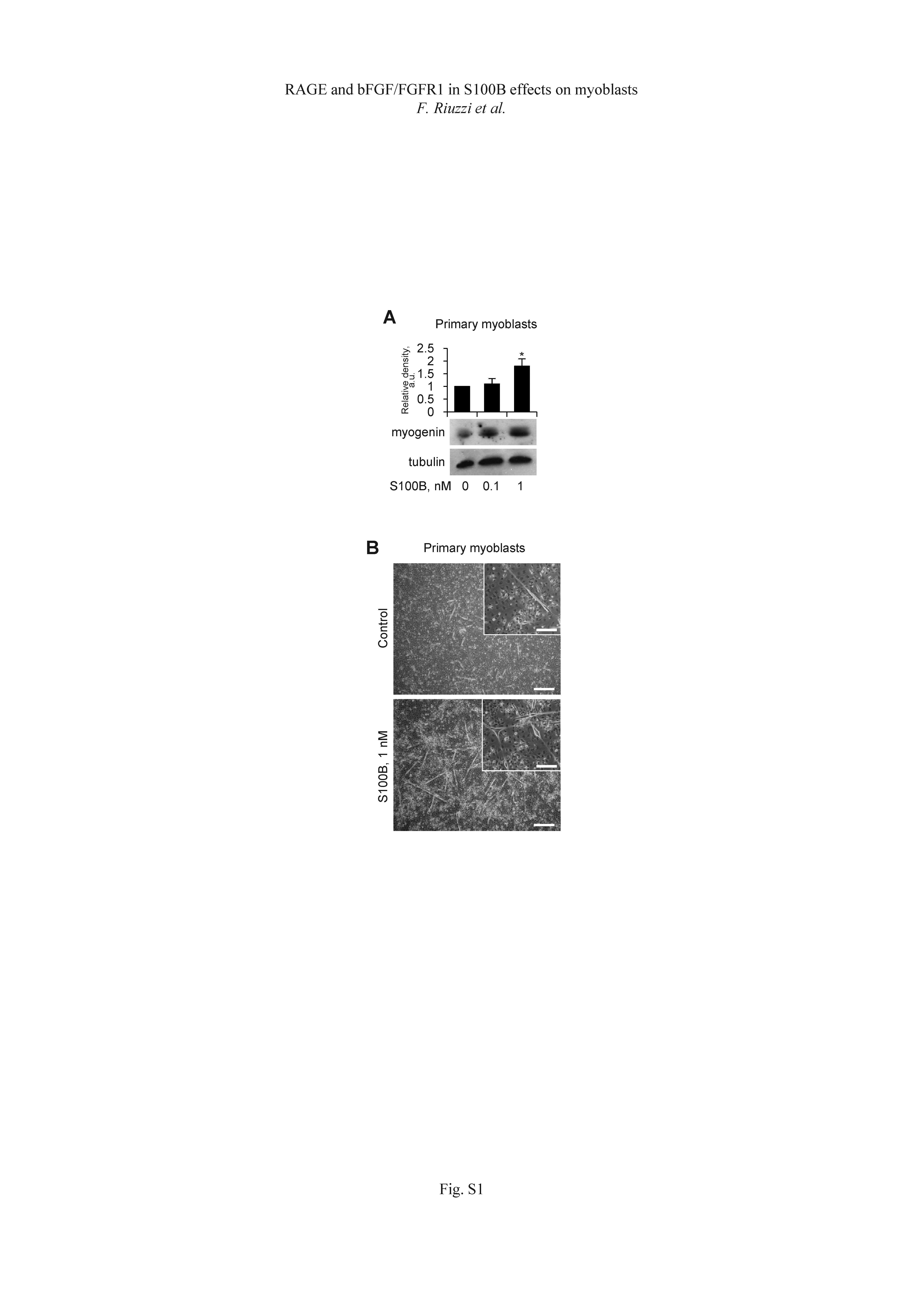

Supplement: Figure S1 — Short-term treatment of LD mouse primary myoblasts with S100B results in enhanced myogenin expression and myotube formation after S100B washout. (A) LD primary myoblasts were cultivated in DM in the absence or presence of S100B for 24 h, washed and cultivated for another 24 h in DM with no additions. Myoblasts were lysed and cell lysates were subjected to Western blotting for detection of myogenin. (B) Same as in A except that LD myoblasts were viewed by phase-contrast at 48 h after washout. Bars = 250 µm (100 µm in insets). (TIF) [file pone.0028700.s001.tif]
